# A Case Report on Prosopometamorphopsia

**DOI:** 10.1192/bjo.2025.10788

**Published:** 2025-06-20

**Authors:** Isuri Wimalasiri, Nimesha Subasinghe, Chathurie Suraweera

**Affiliations:** 1 University Psychiatry Unit, National Hospital of Sri Lanka, Colombo, Sri Lanka; 2 Faculty of Medicine, General Sir John Kotelawela Defence University, Colombo, Sri Lanka; 3 Faculty of Medicine, University of Colombo, Colombo, Sri Lanka

## Abstract

**Aims:** Prosopometamorphopsia is an extremely rare phenomenon where the individual perceives facial distortions in self or others. “Demon face syndrome” and “Alice in Wonderland syndrome” are some lay terms coined to describe this condition.

**Methods:** We report a case of a male in his thirties, married, and a father of one, who presented to the psychiatry unit perceiving distorted facial features of self (autoprosopometamorphopsia) for eight years with exacerbation since last year. He alluded to his reflection in the mirror as the face of a demon. This resulted in significant social avoidance and rumination of other people’s perceptions of his appearance. At the time of presentation, he was having a severe depressive episode with significant occupational and functional decline and was consuming excessive alcohol as a maladaptive coping strategy.

When the symptom first appeared eight years ago while working in the military, he perceived similar distortions in a co-officer’s face in addition to his face, which culminated in him resigning from the forces due to the distress. He did not seek medical treatment, and symptoms went into spontaneous remission after a few months. He was satisfactorily functioning until a year ago.

Apart from prosopometamorphopsia, he did not have any other psychotic symptoms or symptoms suggestive of manic episodes. He was diagnosed with anti-NMDA encephalitis one year ago and had suffered seizures. He reportedly lost the neuroimaging and treatment records related to this episode.

**Results:** The patient was initially diagnosed of Body Dysmorphic Disorder (BDD) and had been treated with adequate trials of antidepressants with very poor response. Due to the severity of depression, functional decline and previous poor response to antidepressants, he was treated with electroconvulsive therapy (ECT) during his current admission. During the one-month post-discharge evaluation, he showed satisfactory improvement and was continued on imipramine 150 mg and olanzapine 20 mg in the night. He was also on topiramate 50 mg nocte from the neurology clinic.

**Conclusion:** Existing literature reveals that prosopometamorphopsia has an organic basis with malfunctioning brain facial recognition systems playing a critical role in its manifestation. In this patient, a history of NMDA encephalitis suggests neuropsychiatric aetiology. Lack of response to conventional treatment for BDD and good response to ECT indicates that this condition is a different entity warranting tailored treatment.

